# Iron stores in pregnant women with sickle cell disease: a systematic review

**DOI:** 10.1186/s12884-020-03326-8

**Published:** 2020-10-16

**Authors:** Desmond Aroke, Benjamin Momo Kadia, Tsi Njim

**Affiliations:** 1 Health and Human Development (2HD) Research Network, Douala, Cameroon; 2 Green Fingers, Buea, Cameroon; 3 Faculty of Epidemiology and Population Health, London School of Hygiene and Tropical Medicine, London, UK

**Keywords:** sickle cell disease, Iron deficiency, Pregnancy, systematic review

## Abstract

**Background:**

Gradual improvements in the management of sickle cell disease (SCD), have led to an increase in the number of women with SCD who reach the age of procreation. However, evidence on the iron status of pregnant women with sickle cell disease (PWSCD) remains inconclusive. We conducted the first systematic review on the prevalence, determinants and maternal/foetal outcomes of iron deficiency anaemia among PWSCD.

**Methods:**

We searched MEDLINE, EMBASE, Global Health, Africa Index Medicus, the Cochrane library databases and reference lists of retrieved publications for studies describing the iron status of PWSCD. The literature search was done over a period of 1 month, with no language or date restrictions applied. Data were extracted on a Microsoft excel sheet. Two authors assessed all included studies for methodological quality and risk of bias.

**Results:**

A total of 710 reports were identified for title and article screening. Five retained studies were conducted before or during the 90s and included 67 participants. After quality assessment, the observational studies were designated to have a “fair” quality assessment while the randomised control trial had an “unclear” quality assessment. The prevalence of iron deficiency anaemia among PWSCD varied by study design and diagnostic method. The overall prevalence ranged from 6.67–83.33%. None of the studies provided evidence on factors associated with iron deficiency anaemia and the randomized trial reported no difference in outcomes between PWSCD who had iron supplementation and those who did not.

**Conclusion:**

Evidence on factors associated with iron deficiency anaemia among PWSCD and maternal/foetal outcomes in PWSCD who have iron deficiency anaemia is poor. The studies included in this review suggests that iron deficiency anaemia may be highly prevalent in PWSCD but due to the very small sample sizes and varied study designs, this evidence is inconclusive. The review shows that there is a need for more studies with robust designs and adequate sample sizes to assess the disease burden of iron deficiency anaemia in PWSCD.

## Background

Sickle cell disease (SCD) consists of a group of inherited red blood cell disorders with at least one sickle cell “S” gene and a non “A” gene [1]. SCD types include HbSS, HbSC, HbS β thalassemia, HbSD, HbSE and HbSO [2]. The greatest burden of SCD is seen in low and middle income countries where lack of resources limits the management of these patients [3].

Over the years, advancements in research and in-depth understanding of SCD have led to improved care of people with SCD. More women with the disease are reaching the reproductive age. Pregnancy in women with sickle cell disease tends to be associated with poor maternal (maternal mortality (pooled OR 10.91, 95% CI 1.83–65.11)) and foetal outcomes (intrauterine growth restriction (pooled OR 2.79, 95% CI 1.85–4.21), perinatal mortality (pooled OR 3.76, 95% CI 2.34–6.06)) [4]. Maternal mortality in a previous report was shown to be about 29 times higher in pregnant women with sickle cell disease (PWSCD) when compared to pregnant women without sickle cell disease in low and middle income countries [5]. Critical review of modifiable factors that could reduce the morbidity associated with this condition is needed to guide clinical case management.

The low iron body stores among adult females coupled with increased pregnancy iron requirements often put pregnant women at risk of iron deficiency anaemia [6–9]. Iron deficiency anaemia in pregnancy is a known significant contributor to maternal morbidity and mortality. Daily iron supplementation in pregnancy is recommended by the World Health organization (WHO) as a proactive measure to reduce anaemia and its associated complications in pregnancy [10]. However, there are no clear guidelines on iron supplementation in the PWSCD subpopulation. In SCD, chronic haemolysis leads to recurrent transfusions and a risk of iron overload [11]. This risk of iron overload amongst SCD patients and risk of iron deficiency in pregnancy makes supplementation of iron in PWSCD a difficult decision. Previous studies have been done to evaluate iron stores amongst PWSCD with varying outcomes [12–16]. While some studies report adequate stores, others have reported deficiency and even depletion [17, 18]. Our study objectives were (1) to estimate the prevalence of iron deficiency anaemia among pregnant women with SCD. (2) To assess socio-demographic, obstetric and clinical factors associated with iron deficiency anaemia among pregnant women with SCD. (3) To evaluate foetal and maternal outcomes among pregnant women with SCD who are iron-deficient.

## Methods

The protocol for this review was registered with PROSPERO (registration number CRD42018109803) and published in a peer reviewed journal [19]. The review is reported following the Preferred Reporting Items for Systematic review and Meta-Analysis (PRISMA) guidelines [20].

We searched the following databases; MEDLINE, EMBASE, Global Health, Africa Journal Online (AJOL), Africa Index Medicus, and the Cochrane library for studies carried out from inception to October 15th 2019. Literature search was done for a period of 1 month (September 15th to October 15th 2019. We used the search strategy and search terms published in the protocol for this study, [19] available on (Additional file 1: Appendix 1). Additionally, we searched the reference lists of eligible studies for article titles with potentially similar objectives. We applied no language restrictions. We aimed to assess the prevalence, associated factors and maternal/foetal outcomes of iron deficiency anaemia among PWSCD. We included all observational studies and clinical trials which provided answers to at least one of the 3 objectives on the iron status in PWSCD as we previously reported [19]. Case reports, commentaries, reviews, editorials, letters, and protocols were excluded.

### Study screening

Two investigators (DA and BMK) performed independent literature searches. After the initial database search, the available data was de-duplicated. DA and BMK independently reviewed titles and abstracts of the studies obtained for eligibility. Each investigator produced a list of potentially eligible studies, and the lists were harmonised into a single list. DA and BMK then independently assessed the full texts of the harmonised list of potentially eligible studies for inclusion. Once more, their findings were harmonised and a comprehensive list of included studies was produced. A third investigator – TN, arbitrated during disagreements between DA and BMK. For publications with ambiguous data, the authors were contacted by email for clarity. For articles without full texts that were potentially eligible for inclusion, the authors were contacted via email or other platforms such as ResearchGate. Constant weekly reminders were sent to these authors and the studies were automatically excluded if no response was received after 1 month [18, 21].

**Fig. 1:**
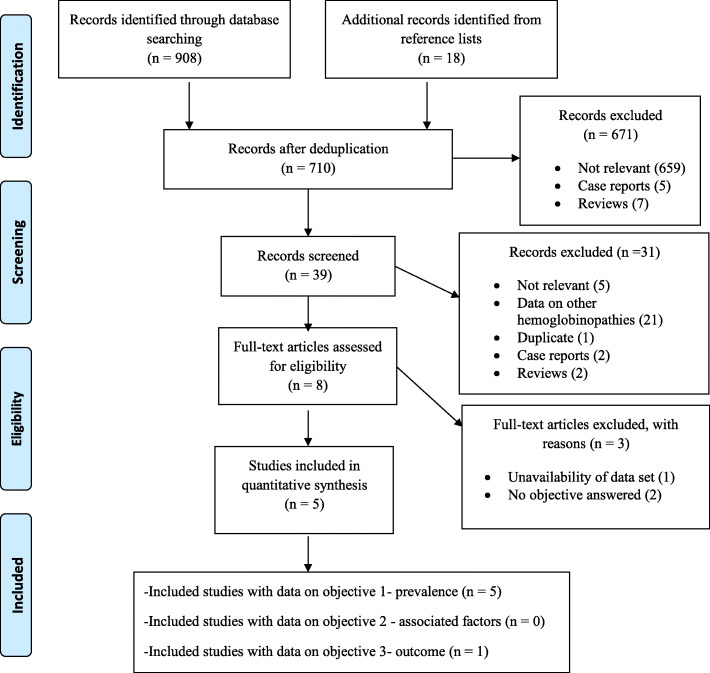
PRISMA P flow diagram

Potentially eligible studies excluded were documented with the various reasons for exclusion. A detailed PRISMA flow chart was used to depict the selection process as shown in Fig. 1.

### Risk of bias and quality assessment

Two reviewers (DA and BMK) independently assessed the methodological quality and the risk of bias for each study. Assessment was done using the Quality Assessment Tool for Observational Cohort and Cross-Sectional Studies of the National Health Institute/National Heart, Lung, and Blood Institute (Additional file 1: Appendix 2) for the observational studies and the Cochrane Risk of Bias Tool for Randomized Controlled Trials (Additional file 1: Appendix 3 and 4) for the randomized control trial [13].

### Data extraction, synthesis and analysis

A data extraction sheet produced on Microsoft excel and pretested by investigators was used to extract the data from selected studies. The following data were extracted; socio-demographic information (country of study population and study setting), study characteristics (the name of the first author, year of publication, study design and setting, mean age, sickle cell genotype, gestational age distribution, transfusion history, sample size and method used to assess body iron stores) and study findings (iron status, prevalence of iron deficiency anaemia, intervention and the outcome of the foetus and mother). The abstracted data was recorded on Microsoft excel 2017 spread sheet. Only five articles were retained for full text review. These articles had varied study designs and very small sample sizes. More so, the studies included women at varying gestational ages and different biomarkers were used to assess iron stores With such heterogeneity in only 5 studies, performing a meta-analysis was not justified. A descriptive approach was thus adopted for data analysis and synthesis. Prevalence of iron deficiency anaemia among PWSCD was sorted generally, by method of diagnosing iron deficiency and by study design. Prevalence was described for each category. Determinants of iron deficiency anaemia were not assessed as none of the 5 articles studied this objective. Information on the maternal/foetal outcomes of iron deficiency anaemia among PWSCD was described as only one study assessed this objective.

### Patient and public involvement

No patient involved.

## Results

The initial literature search yielded 908 articles and 18 additional articles were identified from the reference lists of eligible studies. A total of 5 studies were retained following de-duplication and exclusion of studies not meeting inclusion criteria. The five retained studies had a total of 67 participants. A flow diagram showing the identification and selection of eligible studies is provided on Fig. 1.

### Study characteristics and risk of bias assessment

All studies were hospital-based, single centre studies done in tertiary healthcare facilities. The studies were conducted from 1972 to 1997 and were conducted in 3 countries across 3 continents. Four studies were observational (3 cross sectional [22–24] and 1 case control [14]) and one was a randomized control trial [13]. For the randomized control trial, fourteen pregnant women with sickle cell disease (11 Haemoglobin SS and 3 Haemoglobin SC) were randomized into one of two arms to receive routine antenatal supplementation with ferrous gluconate or with identical placebo tablets. Their haemoglobin levels and bone marrow iron content were measured prenatally and at 6 weeks postpartum. The birth weights and the number of pain crisis in both groups were also recorded [13]. The case control study evaluated Iron stores using transferrin saturation, total iron binding capacity, serum ferritin and bone marrow stainable iron in 22 pregnant and 18 non-pregnant women with SCD. Only the latter was used for this study to estimate prevalence as only mean values were provided for transferrin saturation, total iron binding capacity and serum ferritin. The mean gestational age of participants was 23.3 weeks [14]. Roopnarinesingh evaluated iron stores using bone marrow staining for 6 Negro women with singleton pregnancies in a Spanish General Hospital. Their mean gestational age was 28.7 weeks and none of the participants had been transfused recently [23]. In Jamaica, Anderson assessed the iron stores of 15 PWSCD with a mean gestational age of 16.9 weeks using total iron binding capacity, serum iron and bone marrow stainable iron [22]. None of the 5 studies reported the study period and the study duration. Iron stores were assessed using serum iron (*n* = 2), serum ferritin (*n* = 1), total iron binding capacity (*n* = 1) and bone marrow stainable iron (*n* = 4). Aken’ova et al. used both serum iron and serum ferritin and was the only study that did not use bone marrow stainable iron [24]. The observational studies had a fair risk of bias while the interventional study had mostly an unclear determination of bias risk. A summary of study characteristics and bias assessment is provided on Table 1.

**Table 1:** Study characteristics and risk of bias assessment

Author (year), country	Objective studied	Mean Age	Mean Gestational age	Study design	Recent Transfusion (< 6 months)	Hemoglobin genotype (number of participants)	Sample size	Diagnostic method	Diagnostic criteria	Participants with IDA	Prevalence	Bias
Akinyanju et al. (1987), Nigeria [13]	1,3	22.8	NA	Randomized control trial	None	SS (11) SC (3)	14	Bone marrow iron stain	Grade 0–1/5	3	21.43 ± 4.49	Selection bias: unclear Reporting bias: unclear Performance bias: unclear Detection bias: unclear Attrition bias: Low
Anderson et al. (1972), Jamaica [22]	1	25.27	16.93	Cross sectional study	None^a^	SS (7) SC (6) S-thalassemia (2)	15	Bone marrow iron stain Serum iron TIBC^b^	Grade 0–1/3 < 50 ng/dl < 240 μg/dl	10 1 1	66.67 ± 15.94 6.67 ± 0.87 6.67 ± 0.87	Fair
Oluboyede et al. (1980), Nigeria [14]	1	NA	23.3	Case control study	None	SS (10) SC (12)	22	Bone marrow iron stain	Grade 0–1/3	14	63.34 ± 12.78	Fair
Roopnarinegnh (1976), Spain [23]	1	NA	28.67	Cross sectional study	None	SS (5) SF (1)	6	Bone marrow iron stain	Negative & weakly positive	5	83.33 ± 25.04	Fair
Aken’ova et al. (1977), Nigeria [24]	1	NA	NA	Cross sectional study	NA	SS	10	Serum ferritin Serum iron	< 150 ng/dl < 50 ng/dl	3 3	30 ± 8.52 30 ± 8.52	Fair

^a^Three patients had been transfused at least once; 5 years, 3 years and 6 months prior to the pregnancy, ^b^Total iron binding capacity, NA: not provided in study, IDA; iron deficiency anaemia, SS; Haemoglobin SS, SC; Haemoglobin SC, SF; Haemoglobin SF, Prevalence is reported with ± confidence intervals

### Prevalence of iron deficiency among pregnant women with sickle cell disease

The prevalence of iron deficiency anaemia among PWSCD ranged from 6.67 ± 0.87–83.33 ± 25.04%. Prevalence varied across study design (cross sectional (6.67 ± 0.87–83.33 ± 25.04%) [22–24], case control (63.34 ± 12.78%) [25] and randomized control trial (21.43 ± 4.49%) [13]) and by diagnostic method of iron stores (serum iron (6.67 ± 0.87 & 30 ± 8.52%), serum ferritin (30 ± 8.52%), total iron binding capacity (6.67 ± 0.87%) and bone marrow stainable iron (21.43 ± 4.49% – 83.33 ± 25.04%)). For the case control study, fourteen of the 22 pregnant women (63.6%) and 9 of the 18 non-pregnant women (50%) had scanty or no iron in the bone marrow; the serum ferritin levels increased progressively with greater amount of haemosiderin in the bone marrow. Anderson et al. using total iron binding capacity, serum iron and bone marrow stainable iron had prevalence of 6.67,6.67 and 66.7% respectively [22] Details on the sample size, number of participants with iron deficiency anaemia and prevalence in each category is available on Table 1.

### Factors associated with iron deficiency among pregnant women with sickle cell disease

None of the studies included in this review provided information on factors associated with iron deficiency anaemia among PWSCD.

### Outcomes (foetal and maternal) of pregnant women with SCD who are iron deficient

Only one study followed women through to the postpartum period and provided outcome data [13]. Following initial iron store measurements, all women with SCD were randomized to receive iron gluconate or an identical placebo. At birth, birth weights of the children were measured and at 6 weeks postpartum repeat measurements of the iron stores of the women were done. Three of the 14 participants with low antenatal iron stores did not have any change in their postpartum iron stores in both the trial and control groups. The placebo group showed an aggregate loss of 4 grades of iron repletion in the post natal period while the iron supplemented group showed an aggregate gain of 2 grades. There were no significant differences between the birth weight or the incidence of pain crises in both groups. All three iron deficient women had normal foetal birth weights [13].

## Discussion

This systematic review summarizes data from published studies reporting on iron stores of PWSCD. There is scarcity of studies assessing the disease burden of iron deficiency anaemia in PWSCD.

The studies included in this review may suggest that iron deficiency anaemia is common in PWSCD. Iron deficiency anaemia among pregnant women has previously been reported to be 38.2% on a global scale [26]. Due to complications associated with iron deficiency anaemia in pregnancy, the WHO has recommended routine iron supplementation for pregnant women in countries with a prevalence that exceeds 40% [27]. It is possible that this criterion may be applied to certain sub-populations such as PWSCD. However, the varied study designs, the fact that most of these studies were conducted decades ago, different biomarkers used to assess iron stores and most of all the very small sample sizes makes it difficult for meaningful conclusions to be drawn. Recurrent transfusions are the most common cause of high iron stores in people with SCD [16, 28, 29]. None of the participants in these studies had recently been transfused. This could in part explain why the prevalence of iron deficiency anaemia in these study participants was similar to that of pregnant women without sickle cell disease.

This review included just five studies, with varied study designs and questionable internal and external validity of each. For instance, the cross-sectional study by Roopnarinegnh with 6 participants that were selected using non-probabilistic sampling in a small immigrant population gives prevalence of 83.3% [23] is very likely to have both low internal and external validity. Likewise, the case-control study by Oluboyede and colleagues in a study population of 22 participants with unmatched control groups and a prevalence of 63.3% similarly had low internal validity and should not be primed over a well conducted cross-sectional study. However, the more robust randomized clinical trial which included 14 participants with a prevalence of 21.4% may suggest a more reliable estimate for inference. Before the twenty-first century, a few studies were done to assess the iron stores of PWSCD [13, 23–25, 30]. With improved management strategies on SCD, more people with the disease are becoming pregnant [31]. One will therefore expect more studies looking at this potentially harmful complication of pregnancy in this subpopulation. Unfortunately, in the twenty-first century there is still no evidence based guideline outlining iron supplementation in this subgroup and only one study in 2012 has looked into iron stores in this subpopulation [32]. One could attempt to justify this with the notion of individuality of care (measuring iron status for each pregnant woman with sickle cell disease prior to making decisions on supplementing iron). However, this approach fails to provide a global consensus.

We intended to do a meta-analysis; however the articles gotten from the search were few, had varied study designs and very small sample sizes. More so, the studies were done at different trimesters, different methods were used to assess iron stores and the primary aims of these studies were not to evaluate iron deficiency. With this heterogeneity, performing a meta-analysis was not justified.

None of the five papers studied factors associated with iron deficiency in the sickle cell disease pregnant subpopulation. At least 80% of the participants in these studies were in low- and middle-income countries where iron deficiency could be explained by several sociocultural and health related factors [33–35]. A high prevalence of hookworm infestations, malaria parasitaemia, and low dietary iron intake are potential causes of iron deficiency [33, 34]. Age, gestational age, transfusion history and socioeconomic status are other potential associated factors which could be assessed in future studies. In all the studies however, no participant had been transfused blood recently and no participant was taking iron supplements.

Iron deficiency in pregnancy is known to have several adverse maternal and foetal outcomes [36]. In the papers reviewed in this study, only one article provided information on the outcome of iron deficiency anaemia in the pregnant women with sickle cell disease. Though describing normal foetal birth weights for all participants who had low iron stores, only a very small number (03) of the participants in the study had iron deficiency anaemia. Inferences cannot be made from such a small sample. Akinyanju and colleagues also described postpartum iron stores for pregnant women in both groups of their trial. There was no net change in iron stores both iron supplemented and placebo groups was not accounted for [13]. Again the few participants reported in this study provide little or no evidence on the maternal outcome of iron deficiency in pregnancy. The review has shown that evidence is lacking and there is urgent need for studies with better study designs and larger sample sizes to be carried out.

There are various stakeholders involved in the management of iron deficiency anaemia and at the end of the review, we have realized that we have not answered all the research questions and that evidence on the prevalence remains inconclusive. Consequently, our main recommendations to researchers, academics, and clinicians (who were eagerly waiting for an answer from this review in order to know what to do henceforth) and perhaps, funders (who are not necessarily policy makers but play a role in deciding what research to fund and may be if more funding had been made available to assess iron stores in pregnant women with sickle cell disease, there wouldn’t exist such sobering gap in evidence). More research should be tailored towards identifying which of the biomarkers better quantifies iron deficiency anaemia in this population, what are the foetal and maternal outcomes during iron deficiency anaemia and would iron supplementation curb this burden. We advocate for more studies with larger sample size, using robust study designs such as case control studies, prospective cohort studies and randomized clinical trials to evaluate iron stores in pregnant women at various trimesters. This research will form the basis for recommendations and guidelines on iron supplementation in PWSCD whose iron stores cannot be assessed should be drawn.

### Strengths and limitations of this study

- Globally, there is paucity of data on the iron status of pregnant women with sickle cell disease. This is the first review to summarise published data on the iron status of pregnant women with sickle cell disease.
- The risk of bias was low as an independent review process was done. However, our study estimates are subject to several data limitations.
- The review included various study designs; there is a potential risk of heterogeneity in the results.
- Most papers in this study were done on people of African descent, living in resource-limited settings. Given the studies included in the review, external validity is of major concern. Thus, the observed prevalence may not reflect reality in other ethnic groups and settings. However this population has the highest burden of sickle cell disease.

## Conclusions

Over the years, there have been controversies on the iron status of PWSCD and on the evidence regarding the role of iron supplementation in PWSCD and the associated pregnancy outcomes. We aimed to summarize existing data on this issue through a comprehensive review of available articles on iron status of PWSCD. This suggests that the prevalence of iron deficiency anaemia may vary from very low to very high in PWSCD but due to the very small sample sizes and varied study designs, this evidence is inconclusive. The review shows that there is a need for more studies with robust designs and adequate sample sizes to assess the disease burden of iron deficiency anaemia in PWSCD. Future directions in research could include; iron metabolism in PWSCD, larger sample size and interventional studies on iron status of PWSCD, and iron status by trimester/gestational age of PWSCD.

## Supplementary information

**Additional file 1 Appendix 1.** Search strategy for MEDLINE and adaptability to other databases. **Appendix 2.** Quality Assessment Tool for Observational Cohort and Cross-Sectional Studies. **Appendix 3.** Cochrane Risk of Bias Tool - Cochrane Collaboration modified tool for assessing risk of bias for RCT’s, PART I. **Appendix 4.** Cochrane Collaboration modified tool for assessing risk of bias for RCT’s, PART II.

## Data Availability

Not applicable.

## Abbreviations

SCD: Sickle cell disease
WHO: World health organization
PRISMA: Preferred reporting items for systematic review and meta-analysis
PWSCD: Pregnant women with sickle cell disease
