# Supplementary material for: Iron stores in pregnant women with sickle cell disease: a systematic review
Source: BMC Pregnancy Childbirth. 2020 Oct 16;20:627. doi: 10.1186/s12884-020-03326-8 (PMC7566031; doi:10.1186/s12884-020-03326-8)
Supplement: Supplementary file 1 — Additional file 1 Appendix 1. Search strategy for MEDLINE and adaptability to other databases. Appendix 2. Quality Assessment Tool for Observational Cohort and Cross-Sectional Studies. Appendix 3. Cochrane Risk of Bias Tool - Cochrane Collaboration modified tool for assessing risk of bias for RCT’s, PART I. Appendix 4. Cochrane Collaboration modified tool for assessing risk of bias for RCT’s, PART II. [file 12884_2020_3326_MOESM1_ESM.docx]

**Appendix**

**Appendix 1: Search strategy for MEDLINE and adaptability to other databases**

| Searches | Search combinations | Search terms |
| --- | --- | --- |
| S1 |  | (MH "Anemia, Sickle Cell+") OR (MH "Sickle Cell Trait") |
| S2 |  | “Sickle cell anaemia” OR “sickle cell anemia” OR “sickle cell trait” OR “Sickle cell disease” OR “sickle cell haemoglobinopathy” OR “haemoglobinopathy” OR “hemoglobinoathy” OR “abnormal haemoglobin” OR “abnormal haemoglobin” OR “sickler” OR “sicle cell” OR “Drepanocytosis” OR “HbSS” OR “HbSC” OR “SCD” OR “SS” OR “SC” |
| S3 | S1 OR S2 |  |
| S4 |  | (MH "Pregnancy+") OR (MH "Pregnancy Outcome+") OR (MH "Pregnancy Trimesters+") OR (MH "Pregnancy Complications+") |
| S5 |  | “Pregnan*” OR “pregnancy outcome” OR “pregnancy trimesters” OR “pregnancy complications” OR “Gestation*” OR Pregnant OR “Gestation age” OR “gravid*” OR “Expect* mother” OR “trimester” OR “parity” |
| S6 | S4 OR S5 |  |
| S7 |  | (MH "Iron+") OR (MH "Iron, Dietary") OR (MH "Iron Overload+") OR (MH "Dietary Supplements+") OR (MH "Anemia, Iron-Deficiency") |
| S8 |  | Iron OR “diet* iron” OR “Iron overload” OR “dietary supplement*” OR “iron deficiency anaemia” OR “iron deficiency anemia” OR ”iron status” OR “iron stores” OR “iron supplementation” OR “serum iron” OR “iron deficiency” OR “serum ferritin” OR “bone marrow stainable iron” OR “total iron binding capacity” OR “transferrin” OR “iron overload” OR “microcytic anaemia” OR “microcytic anemia” OR “anaemia” OR “anemia” OR OR “low body iron” OR “body iron” OR “low serum iron” OR “high serum iron” OR “high body iron” OR “normal serum iron” OR “normal body iron” OR OR “blood iron” OR “iron indices” OR “body iron indices” OR “serum iron indices” OR OR “ferritin” |
| S9 | S7 OR S8 |  |
| S10 | S3 AND S6 AND S9 |  |

| **Criteria** | **Aken’Ova et al** | **Anderson et al** | | **Oluboyede et al** | | **Roopnarinesingh et al** | |
| --- | --- | --- | --- | --- | --- | --- | --- |
| 1. Was the research question or objective in this paper clearly stated? | Y | Y | | Y | | Y | |
| 2. Was the study population clearly specified and defined? | Y | Y | | Y | | Y | |
| 3. Was the participation rate of eligible persons at least 50%? | CD | CD | | CD | | CD | |
| 4. Were all the subjects selected or recruited from the same or similar populations (including the same time period)?  Were inclusion and exclusion criteria for being in the study pre-specified and applied uniformly to all participants? | CD  CD | CD  CD | | CD  Y | | CD  Y | |
| 5. Was a sample size justification, power description, or variance and effect estimates provided? | N | N | | N | | N | |
| 6. For the analyses in this paper, were the exposure(s) of interest measured prior to the outcome(s) being measured? | NA | NA | | NA | | NA | |
| 7. Was the timeframe sufficient so that one could reasonably expect to see an association between exposure and outcome if it existed? | NA | NA | | NA | | NA | |
| 8. For exposures that can vary in amount or level, did the study examine different levels of the exposure as related to the outcome (e.g., categories of exposure, or exposure measured as continuous variable)? | NA | NA | | NA | | NA | |
| 9. Were the exposure measures (independent variables) clearly defined, valid, reliable, and implemented consistently across all study participants? | Y | Y | | Y | | Y | |
| 10. Was the exposure(s) assessed more than once over time? | N | N | | N | | N | |
| 11. Were the outcome measures (dependent variables) clearly defined, valid, reliable, and implemented consistently across all study participants? | NA | NA | | NA | | NA | |
| 12. Were the outcome assessors blinded to the exposure status of participants? | NA | NA | | NA | | NA | |
| 13. Was loss to follow-up after baseline 20% or less? | NA | NA | | Y | | NA | |
| 14. Were key potential confounding variables measured and adjusted statistically for their impact on the relationship between exposure(s) and outcome(s)? | N | N | | N | | N | |
| Quality Rating (Good, Fair, or Poor) (see guidance) | F | F | | F | | F | |
| Rater #1 initials: DA |  |  |  |  |  |  |  |
| Rater #2 initials: BMK |  |  |  |  |  |  |  |
| Additional Comments (If POOR, please state why): |  |  |  | |  | |  |

**Appendix 2: Quality Assessment Tool for Observational Cohort and Cross-Sectional Studies**

Developed by the National Heart, Lung and Blood Institute (NHLBI)

*CD, cannot determine; NA, not applicable; NR, not reported

**Appendix 3: Cochrane Risk of Bias Tool - *Cochrane Collaboration modified tool for assessing risk of bias for RCT’s, PART I***

| Using the guidance provided at the end of this form, select either “high”, “low” or “unclear” for each judgment. When complete, proceed to **Part II of the Quality Assessment Form REF ID:** | | | | | |
| --- | --- | --- | --- | --- | --- |
| **Domain** | **Description** | **High risk of bias** | **Low risk of bias** | **Unclear risk of bias** | **Reviewer Assessment** |
| *Selection bias*  ***Random sequence generation*** | Described the method used to generate the allocation sequence in sufficient detail to allow an assessment of whether it should produce comparable groups.  **Reviewer Comments:** No detailed description of allocation of participants | Selection bias (biased allocation to interventions) due to inadequate generation of a randomized sequence. | Random sequence generation method should produce comparable groups | Not described in sufficient detail | **Judgement**  **Random sequence generation**  □ **Unclear** |
| *Selection bias*  ***Allocation concealment*** | Described the method used to conceal the allocation sequence in sufficient detail to determine whether intervention allocations could have been foreseen in advance of, or during, enrolment.  **Reviewer Comments:** No detailed description of concealment of the iron and placebo participants | Selection bias (biased allocation to interventions) due to inadequate concealment of allocations prior to assignment. | Intervention allocations likely could not have been foreseen in advance of, or during, enrolment | Not described in sufficient detail | **Judgement**  **Allocation concealment**  □ **Unclear** |
| *Reporting bias*  ***Selective reporting*** | Stated how the possibility of selective outcome reporting was examined by the authors and what was found.  **Reviewer Comments:** Authors did not describe reporting any specific outcomes | Reporting bias due to selective outcome reporting. | Selective outcome reporting bias not detected | Insufficient information to permit judgement (*It is likely that the majority of studies will fall into this category.)* | **Judgement**  **Selective reporting**  □ **Unclear** |
| *Other bias*  ***Other sources of bias*** | Any important concerns about bias not addressed above. If particular questions/entries were pre-specified in the study’s protocol, responses should be provided for each question/entry.  **Reviewer Comments:** consecutive recruitment of participants; sampling bias | Bias due to problems not covered elsewhere in the table. | No other bias detected | There may be a risk of bias, but there is either insufficient information to assess whether an important risk of bias exists; or insufficient rationale or evidence that an identified problem will introduce bias. | **Judgement**  **Other sources of bias**  □ **High**  □ **Low**  □ **Unclear** |

*Use this form to assess risk of bias for randomized controlled trials*.

Bias is assessed as a judgement (high, low, or unclear) for individual elements from five domains (selection, performance, attrition, reporting, and other).

Risk of selection, reporting, and other bias are assessed in the **Quality Assessment Form Part I.** Risk of performance, detection, and attrition bias are assessed using the **Quality Assessment Form Part II.**

***Appendix 4: Cochrane Collaboration modified tool for assessing risk of bias for RCT’s, PART II***

| Risk of bias for the domains in the Form Part II will be assessed for each main or class of outcomes. Please indicate the specific outcome and complete the assessment for each. **REF ID:** | | | | | |
| --- | --- | --- | --- | --- | --- |
| **Outcomes:** | | | | | |
| **Domain** | **Description** | **High risk of bias** | **Low risk of bias** | **Unclear risk of bias** | **Reviewer Assessment** |
| *Performance bias*  ***Blinding (participants and personnel)*** | Described all measures used, if any, to blind study participants and personnel from knowledge of which intervention a participant received. Provided any information relating to whether the intended blinding was effective.  **Reviewer Comments:** No detailed description of blinding procedure for participants and personnel | Performance bias due to knowledge of the allocated interventions by participants and personnel during the study. | Blinding was likely effective. | Not described in sufficient detail | **Judgement**  **Blinding (participants and personnel)**  □ **Unclear** |
| *Detection bias*  ***Blinding (outcome assessment)*** | Described all measures used, if any, to blind outcome assessors from knowledge of which intervention a participant received. Provided any information relating to whether the intended blinding was effective.  **Reviewer Comments:** No detailed description of outcome blinding of participants | Detection bias due to knowledge of the allocated interventions by outcome assessors. | Blinding was likely effective. | Not described in sufficient detail | **Judgement**  **Blinding (outcome assessment)**  □ **Unclear** |
| *Attrition bias*  ***Incomplete outcome data*** | Described the completeness of outcome data for each main outcome, including attrition and exclusions from the analysis. Stated whether attrition and exclusions were reported, the numbers in each intervention group (compared with total randomized participants), reasons for attrition/exclusions where reported.  **Reviewer Comments:** Data was described for all subjects who met inclusion criteria | Attrition bias due to amount, nature or handling of incomplete outcome data. | Handling of incomplete outcome data was complete and unlikely to have produced bias | Insufficient reporting of attrition/exclusions to permit judgment of ‘Low risk’ or ‘High risk’ (e.g. number randomized not stated, no reasons for missing data provided) | **Judgement**  **Incomplete outcome data**  □ **Low** |

*Use this form to assess risk of bias for randomized controlled trials.*

Bias is assessed as a judgement (high, low, or unclear) for individual elements from five domains of bias (selection, performance, attrition, reporting, and other).

Using the guidance provided at the end of this form, select either “high”, “low” or “unclear” for each judgement.
